# Green Solvents for Extraction of Natural Food Colorants from Plants: Selectivity and Stability Issues

**DOI:** 10.3390/foods13040605

**Published:** 2024-02-16

**Authors:** Milena Tankova Tzanova, Zvezdelina Yaneva, Donika Ivanova, Monika Toneva, Neli Grozeva, Neli Memdueva

**Affiliations:** 1 Faculty of Agriculture, Department of Biological Sciences, Trakia University, 6000 Stara Zagora, Bulgaria; n.grozeva@trakia-uni.bg (N.G.); neli.memdueva.21@trakia-uni.bg (N.M.); 2 Faculty of Veterinary Medicine, Department of Pharmacology, Animal Physiology and Physiological Chemistry, Trakia University, 6000 Stara Zagora, Bulgaria; zvezdelina.yaneva@trakia-uni.bg (Z.Y.); donika.ivanova@trakia-uni.bg (D.I.); monika.toneva@trakia-uni.bg (M.T.); 3 Medical Faculty, Department of Medicinal Chemistry and Biochemistry, Trakia University, 6000 Stara Zagora, Bulgaria

**Keywords:** anthocyanins, betalains, carotenoids, green solvents, plant colorants, selectivity, stability

## Abstract

Consumers associate the color of food with its freshness and quality. More and more attention is being paid to natural colorants that bring additional health benefits to humans. Such natural substances are the carotenoids (yellow to orange), the anthocyanins (red to blue), and the betalains (red and yellow), which are very sensitive to exposure to light, air, high temperatures, and chemicals. Stability and diversity in terms of color can be optimized by using environmentally friendly and selective extraction processes that provide a balance between efficacy, safety, and stability of the resulting extracts. Green solvents like water, supercritical fluids, natural deep eutectic solvents, and ionic liquids are the most proper green solvents when combined with different extraction techniques like maceration, supercritical extraction, and ultrasound-assisted or microwave-assisted extraction. The choice of the right extracting agent is crucial for the selectivity of the extraction method and the stability of the prepared colorant. The present work reviews the green solvents used for the extraction of natural food colorants from plants and focuses on the issues related to the selectivity and stability of the products extracted.

## 1. Introduction

Consumers associate the color of food with its freshness and quality. The coloring of various foods with plant-based additives has been practiced for centuries [1]. The color of plants is due to the presence of byproducts which contain molecules that are able to absorb light selectively. Based on their chromophores, these substances can be divided into chlorophylls (green), carotenoids (yellow to orange), anthocyanins (red to blue), and betalains (red and yellow). Pumpkins make cookies and cakes yellow, tomatoes and beetroots make soups red, spinach makes pane cake green, and blueberries make shake drinks blue–violet, etc. These plant products have not only a specific color but also an aroma and carry the taste of the fruit or vegetable in the meals included. Synthetic colorants bring only color, and in this aspect are universal and suitable for every food. Moreover, the use of such food pigments can lead to the formation of toxic compounds during the production of food additives, which compromise the safety of the food products [2]. In addition, some of them have been associated with allergic reactions, attention deficits in children, and some types of cancer [3]. The Food and Drug Administration (FDA) and European Food Safety Authority (EFSA) organizations regulate the use of food colorants, and after strict tests on their toxicity, they can be involved in the market.

More and more attention is being paid to universal dyes that are of natural origin. When using natural pigments in food production, not only color is obtained. In addition, these compounds also exhibit biological activities that are beneficial to human health, especially in reducing the risk of some chronic diseases [4]. The increase in interest in the application of these natural colorants in the food industry has been stimulated by their health benefits. It is not just color, but bioactivity is also added.

The use of natural colorants is associated with a number of disadvantages. The conventional extraction methods applied in their production are very expensive, not only because of the enormous amounts of solvents, but also as a result of the accumulation of environmentally harmful waste [5]. Less stability and limited diversity in terms of color shade are still problems of natural colorants [6]. The latter drawbacks can be optimized by using environmentally friendly and selective extraction processes that provide a balance between potency, safety, and stability of the resulting extracts. One of the six principles of green extraction is the selection of the proper extraction solvent [7], this is also a very important contributor to the selectivity of the extraction method and the stability of the colorant prepared [8,9]. Several review articles published recently have focused on method conditions and techniques for the extraction of natural colorants from plants [5,9,10,11,12]. The aspect of their selectivity is not discussed. The plant matrix is complex and extraction results in a cocktail of substances. Therefore, the solvents and extraction technique must be carefully selected, which has great importance for the subsequent steps of extract preparation [8]. The selectivity of this first extraction step contributes to the effectiveness, profitability, and simplicity of the entire extract production process. On the other hand, natural pigments are organic compounds with high sensitivity to light, air, high temperatures, and chemicals [9]. It is very important, to ensure the preservation of their properties. In addition, these products are used in food processing, so the responsibility is even greater. The present work reviews the green solvents used for the extraction of natural food colorants from plants and focuses on the issues related to the selectivity and stability of the products prepared.

## 2. Classification of Natural Colorants with Plant Origin and Relevant Sources

Carotenoids, anthocyanins, and betalains are the primary natural pigments used in the food industry because of their positive biological effects, especially in reducing the risk of some chronic diseases [4]. In addition, these natural compounds exhibit antioxidant activity and possess the ability to preserve food [13].

### 2.1. Carotenoids

**Figure 1:**
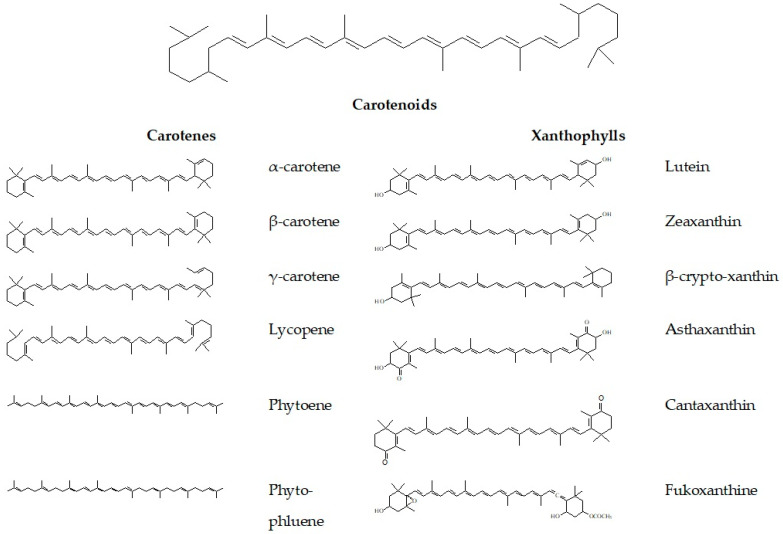
Classification of carotenoids and their representatives.

The base structure of carotenoids is built from eight isoprene molecules, which makes the carotenoids tetraterpene derivatives [14]. This structure is responsible for red, yellow, and orange coloration. The carotenoids can be divided into two subgroups: carotenes and xanthophylls (Figure 1). Carotene molecules consist only of carbon and hydrogen atoms and are definitely fat-soluble. The name “carotene” comes from the word carrot (*Daucus carota*), from which it was isolated by Wachenroder in 1831 [15]. The most common carotenes with commercial potential are α-carotene, β-carotene, and lycopene [16]. In xanthophyll molecules, there are also oxygen atoms in the structure of hydroxyl and carbonyl groups. Therefore, the xanthophylls are more polar as compared to the carotenes. The most common xanthophylls with commercial potential are lutein, asthaxanthin, and cantaxanthin [16].

The European Union regulates carotenoids as food additives under number E 160 [17].

The carotenoid family includes about 700 known structures [18] and is widespread in nature. These natural compounds are synthesized by bacteria (including cyanobacteria) and eukaryotic organisms (algae, fungi, and plants), including phototropic and non-phototropic organisms (except animals). These phytopigments take part in photosynthesis, acting as photoreceptors, and protecting chloroplasts from excess light and from reactive species generated during oxygenic photosynthesis. Therefore, these unique natural compounds provide the most efficient response to photooxidative stress [19]. In addition, humans and mammals use carotenes as raw material in retinol synthesis. These compounds are known as provitamin A carotenoids.

Table 1 presents the plants that are the richest in carotenoids and used as a source for their production. Food processing wastes could also be suitable natural sources for carotene extraction. Microalgae are the proper raw material for xanthophyll production. The most relevant sources for asthaxanthin are the microalgae *Haematococcus pluvialis*, where the yield is more than 2.7–3.8% dry weight [20]. Cantaxanthin is found in sufficient quantities in some fungi, microalgae, and bacteria [21]. The brown algae are a rich source of fucoxanthine [22]. Abdel-Aal et al. proposed that lutein and zeaxanthin are the most common xanthophylls in green leafy vegetables [23].

### 2.2. Anthocyanins (ACNs)

**Figure 2:**
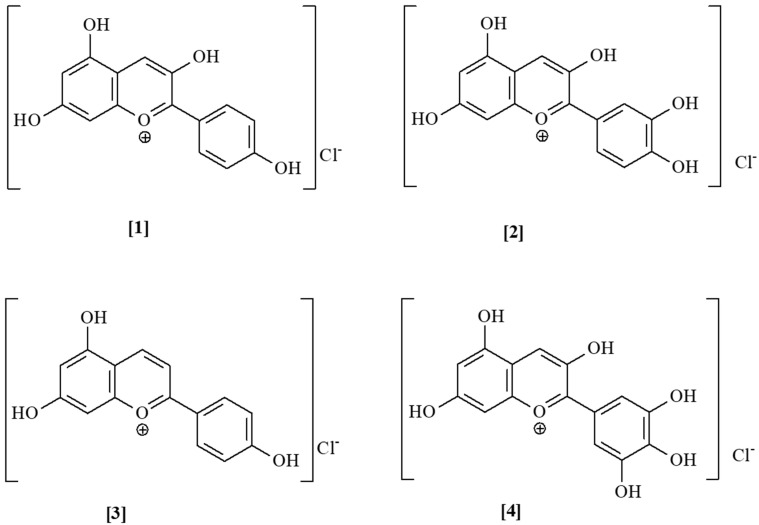
Anthocyanidin structures: Pelargonidin [1], cyanidin [2], apigenidin [3], and delphinidin [4].

ACNs are glucosides of the anthocyanidin group (Figure 2), which belong to the flavonoid family—derivates of flavan. Approximately 700 natural ACNs have been identified [5]. Cyanidin-3-glucoside is the major ACN found in most plants. These natural compounds are water-soluble because of their ionic structure and glycoside group [24]. They are the red and blue dyes of flowers and some fruits, e.g., blueberries, blackberries, grapes, aronia, etc., which are concentrated in the fruit skins [8]. The European Union regulates ACNs as a food additive under number E 163 [17]. Their color depends on their pH: in an acidic medium of pH ≤ 3 ACNs are red (because of a flavilyum cation), while at pH = 6–7, a quinonoid anhydro base is formed, and the color is violet. At a weakly alkaline pH (pH = 7–8) these compounds provide blue coloration, and at a strongly basic pH (pH ≥ 11) they are unstable and decompose into dark brown oxidized compounds [25].

Anthocyanins are characterized by high antioxidant potential. Scientific studies show their protective activities against various non-infectious diseases and confirm their health effects [25].

Appropriate ecological and economical sources of ACNs are food processing waste [26] and edible flowers [27]. Using waste from the food industry is in itself environmentally friendly because the waste is recycled, i.e., it is applied as a raw material for the production of natural food and feed colorants, which are not only safe but also beneficial to human and animal health. Flowers of edible and cultivated plants are economical and come-at-able sources for natural colorant production. A promising source of cyanidin-3-O-glycoside is *Escherichia coli* [28]. These microorganisms produce anthocyans. However, this process has not yet found commercial application because its control parameters have not been developed for a production scale. The most studied among the natural antocyanidins is cyanidin (Table 1).

**Table 1:** Relevant natural source of plant colorants for food.

Colorant Group (Base Structure)	Representative Compounds	Relevant Natural Source	References
Carotenoids	Carotenes		
	β-carotene	Carrot juice processing waste Apricot and shrimp wastes Orange peel Citrus fruits waste	[29] [30] [31] [32]
	Lycopene	Tomato processing waste Pumpkin peels	[33,34] [35]
	Xanthophylls		
	Asthaxanthin Canthaxantin	*Haematococcus pluvialis* *Aspergillus carbonarius* *Botryococcus braunii* *Bradyrhizobium* sp. *Gordonia jacobaea* *Micrococcus roseus*	[14] [36] [37] [38] [39] [40]
	Fucoxanthin Lutein; Zeaxanthin	Brown algae Parsley; Spinach; Red pepper *Heterochlorella luteoviridis*	[21] [41] [42]
Anthocyanins (Anthocyanidins)	Cyanidin	Cherry pomace Raspberry pomace Blackberry pomace Redcurrant pomace Chokeberry pomace Plum skins	[43] [44] [44] [44] [45] [46]
	Pelargonidin	Strawberry pomace	[47]
	Delphinidin	Blueberry pomace Eggplant peels	[48,49] [50]
	Delphinidin; cyanidin	Blackcurrant pomace	[51]
	Cyanidin; peonidin	Cranberry pomace	[52]
	Petunidin	Purple potato peels	[53]
	Delphinidin; cyanidin	Jamaica flower (*Hibiscussabdariffa* L.)	[54]
	Cyanidin	Camellia (*Camellia japonica*) Cornflower (*Centaurea cyanus*) Garden chrysanthemum (*Chrysanthemum Morifolium*) Apple flower (*Malus* spp.) Rose (*Rosa* spp.) French marigold (*Tagetes patula*)	[55] [56] [57] [58] [59] [60]
	Delphinidin	Common violet, sweet violet (*Viola odorata*)	[61]
	Cyanidin; delphinidin	Daylily (*Hemerocallis fulva*) Lilac (*Syringa vulgaris* L.)	[62] [63]
	Malvidin, delphinidin; petunidin; peonidin	Geranium (*Pelargonium* spp.)	[64]
	Cyanidin, malvidin, peonidin	Petunia (*Petunia hybrid*)	[65]
	Cyanidin; malvidin, delphinidin; petunidin; peonidin	Red clover (*Trifolium pretense*)	[66]
	Delphinidin; cyanidin pelargonidin	Tulip (*Tulipa* spp.)	[67]
	Cyanidin; delphinidin, malvidin; petunidin	Pansy (Viola × wittrockiana)	[68]
	Cyanidin, pelargonidin and malvidin	Lavender (*Lavandula angustifolia* Mill.)	[69]
	Cyanidin; peonidin	Magnolia (*Magnolia* spp.) Chinese quince (*Chaenomeles* spp.)	[70] [71]
	Cyanidin; petunidin	Passion flower (*Passiflora incarnate*)	[72]
	Malvidin; pelargonidin; peonidin	Garden balsam, rose balsam (*Impatiens balsamina L*.)	[73]
	Cyanidin; pelargonidin	Saffron (*Crocus sativus*) Dahlia (*Dahlia mignon*)	[74] [75]
	Malvidin; cyaniding; pelargonidin	Tuberous begonia (*Begonia* × *tuberhybrida* *Voss*.)	[76]
	Cyanidin, delphinidin; malvidin; peonidin; pelargonidin; petunidin	Marigold (*Calendula officinalis*)	[77]
Betalains (BLN)	Betacyanins (BCN)	Fruit pulps of *Hylocereus cacti* Dragon fruit peel Chenopodium quinoa willd hulls *Alternanthera brasiliana* Prickly pear peels	[78] [79] [80] [81] [82]
	Betaxanthin (BXN)	Yellow pitaya Prickly pear fruits	[83] [84]
	BCN and BXN	Fruits *Opuntia* spp. *Alternanthera sesillis* Red beet roots Beet leaves Beet pomace *Beta vulgaris* peels Waste red beet stalk Tubers of *Ullucus tuberosus* *Basella alba* Grains of *Chenopodium formosanum* Quinoa grains	[85,86] [87] [88,89] [90] [91] [92] [93] [94] [95] [96] [97]

### 2.3. Betalains (BLNs)

Colored substances called BLNs are derived from betalamic acid and are divided into two subgroups because of their structure: betaxanthins (indicaxanthin, vulgaxanthin I and II) and betacyanins (betanin, isobetanin, neobetanin, and prebetanin). BLNs have proven antioxidant activities [55]. These natural compounds have exhibited promising anti-inflammatory, lipid-lowering, antidiabetic, and anti-obesity effects [98,99].

**Figure 3:**
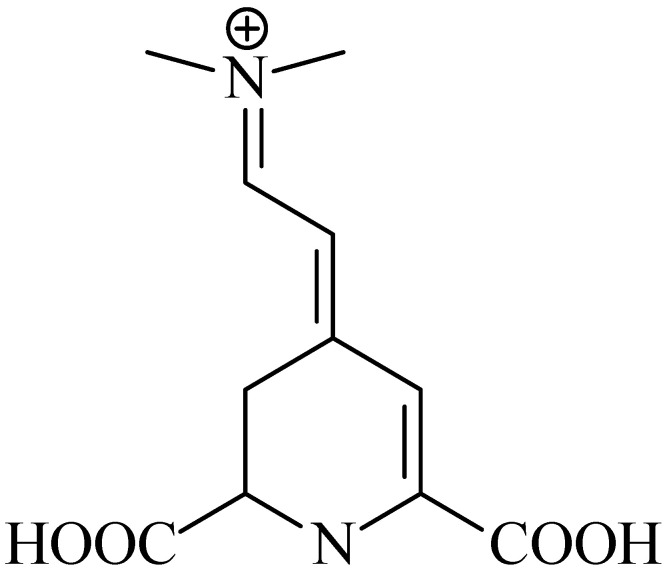
Main betalain chromophore structure.

The bethaxantins are responsible for orange–yellow coloring (λ_max_~480 nm); the betacyanins (betanins) for red–violet coloring (λ_max_~540 nm) [100]. The chromophore structure in BLNs is presented in Figure 3.

Betacyanidins are the aglycones of bethacyanins (BCs) and betanidin is the aglycones of most betacyanins [101]. They are used as food additives. The main representative of BCs is betanidin-5-O-β-glycoside (betanin), the major pigment in red beets [102]. It is known as ‘beetroot red’ and is used in the food industry as additive E162 [17].

The color of BCs depends on pH: red–violet at a pH between 4 and 5, and blue–violet- at a higher pH. At alkaline pH levels, these compounds hydrolyze spontaneously. Betanins are water-soluble. They decompose under exposure to light, heat, oxygen, and the presence of metal ions. Therefore, these pigments are used in frozen products, products with a short shelf life, and products sold in a dry state [103]. Compared to ACNs, betalains have higher solubility in water, exhibit significantly higher color strength, and are generally stable in the pH range of 3–7 [104]. Therefore, they are more suitable for application in slightly acidic and neutral foods than ACNs.

ACNs and BLNs have never been isolated from the same plant [101]. So, this is a good prerequisite for their selective isolation. Plants from the *Caryophyllales* family are good sources of BLNs [105]. They can be found in the roots, fruits, and flowers [106]. The conventional sources of BLNs are red and yellow beetroot (*Beta vulgaris* L. ssp. vulgaris), Swiss chard (*Beta vulgaris* L. ssp. cicla), amaranth (Amaranthus sp.), and some cactus fruits (*Opuntia* and *Hylocereus genera*) [101]. BLNs in cactus fruits cover a broader color spectrum as compared to red beet, from yellow–orange (*Opuntia* sp.) to red–violet (*Hylocereus* sp.). In recent years, new proper sources of BLNs have been found (Table 1).

## 3. Green Solvents

“Green solvents’’ are solvents that minimize the environmental impact of application [107]. To consider a solvent environmentally friendly, its application must not cause health and safety problems, or indirect effects arising from its production, use, and disposal, including the depletion of non-renewable sources [108]. Innocuous and renewable, water and carbon dioxide take the first place in the list of green solvents.

Water is the most popular and greenest solvent ever applied. It is inexpensive and environmentally friendly because of its non-toxicity, non-flammability, and recyclability [109]. Moreover, due to the possibility of changing the physicochemical properties of water with temperature and pressure, the applications of water are even more expanded, for instance, in subcritical extraction processes of less polar compounds [110].

The extraction of bioactive compounds from plant material using supercritical fluids (SFs) takes place at low temperatures, which saves thermo-unstable compounds. There are more advantages: hazardous solvents are avoided, and SFs are easy to remove from the extracted compounds [111]. Commonly, carbon dioxide is applied under supercritical conditions in extraction processes. The wide application of carbon dioxide as a supercritical fluid results mainly from its advantageous supercritical temperature and pressure conditions (31 °C and 73.8 bar), its inexpensiveness, and its environmental friendliness [112].

Surface active agents also should be included in the “green solvents” group. Surfactants are amphiphilic molecules composed of a polar “head” and long hydrophobic “tail”. They reduce the surface tension surrounding the molecules of the insoluble substance and thus make it soluble. These substances have been used for extraction for many years, and a broad spectrum of sample preparation techniques have been developed. After the extraction process, the surfactant-rich phase is separated, e.g., by centrifugation. These techniques are characterized by advantages, such as low cost, nontoxic extractant, simplicity, and high capacity to concentrate a wide range of analytes. Surface active agents are useful for extracting and concentrating small amounts of substances already dissolved in some fluids, such as pesticides or heavy metals in water, antibiotics in biological fluids, etc. They find application in analysis rather than for preparatory purposes [112].

Another class of green solvents is ionic liquids (ILs), which have attracted growing interest due to their unique physicochemical properties. ILs are non-molecular compounds with large organic cations and small inorganic anions, whose melting point is below 100 °C [112]. They have a high solubility degree for a wide spectrum of substances, because of the unique interaction between their ions and the dissolved compounds. The application of ILs as analytical solvents has increased, as they provide a “green” alternative to volatile organic solvents.

An alternative to ILs is deep eutectic solvents (DESs), which have comparable characteristics to ILs but are cheaper to produce due to the lower cost of the raw materials, are less toxic and are often biodegradable [113]. However, the high viscosity and solid state of most DESs at room temperature restrict their application as extraction solvents. Their biodegradability is extraordinarily high, and they are either non-toxic or with very low toxicity. Due to their minimal ecological footprint, low cost of their constituents, tenability of their physicochemical properties, and ease of preparation, DESs are successfully and progressively replacing often hazardous and volatile organic compounds in many fields of science. In addition, these solvents have recycling potential, and the extracted bioactive compounds could be easily purified. The main disadvantage of these solvents is their high viscosity, which leads to mass transfer problems and limits the extraction process. To reduce the viscosity, water is added (5–30%), but this limits their affinity to non-polar compounds [114]. Recently, new types of extraction solvents, natural deep eutectic solvents (NADESs), have been introduced to analytical practice. In addition to all the advantages of DESs, NADESs are even more environmentally friendly due to their natural origin [115].

Bio-derived solvents are solvents that are produced from biomass in a biorefinery and that can be biodegraded after being used [116]. The most appropriate representatives of this group are biodiesels, terpenes, and 2,5-dimethylfuran, which are suitable for the extraction of nonpolar target compounds.

The strongest advantage of the solvents listed is their low environmental impact. They also have quite a few drawbacks. For example, the high production costs for ILs and DESs, high energetic demands, and use of an organic co-solvent for supercritical fluid extraction (SFE). However, if the extraction of natural colorants is selective, the cost of purification and stabilization can be reduced. Furthermore, the subsequent production steps can be avoided.

## 4. Extraction of Natural Colorants Using Green Solvents

Natural colorants are usually extracted from plant sources using diverse techniques after the drying and grinding of the plant material, followed by the selection of a suitable solvent for the target product and subsequent extraction using conventional and modern techniques. The targetted isolated compounds used for coloring are purified using chromatographic or other separation techniques and are characterized by spectral techniques [117]. The right solvent is chosen based on the polarity of the target compound and the subsequent stage of the extraction technique procedure [8]. Using a proper solvent can lead to the achievement of several goals at once: environmental friendliness, selectivity, and economic benefits.

### 4.1. Extraction of Carotenoids

Carotenoids are non-polar substances and, following the maxima “like dissolves like”, they are well soluble in non-polar solvents. Solvents with manufacturing applications are subject to registration, evaluation, authorization, and restriction of chemicals [118]. Vinas-Ospino et al. classified the green solvents used for the extraction of carotenoids from fruit and vegetable byproducts: vegetable oils, terpenes, supercritical fluids (CO_2_), ILs (ammonium salts), and NADESs [119]. Table 2 presents the developed extraction methods for extracting carotenoids from plants for use as food colorants using green solvents.

- Using edible oils

Carotenoids are highly soluble in edible oils, which were often used for their extraction because of their low cost and absence of environmental impact. This extraction has more advantages: the carotenoids are protected against oxidation, and extractant evaporation is not necessary when they are applied as food colorants [13]. The most applied technique is ultrasound-assisted extraction (UAE), and the most widely used oil is sunflower oil. Oleic acid turns out to be a good option, too, because carotenoid-enriched extracts of oleic acid can be directly applied to food products [125]. Good β-carotene sources are carrot and citrus wastes, and a good lycopene source is tomato fruits (Table 2).

Teramukai et al. reported the effective extraction of fucoxanthin from brown seaweed (*Sargassum horneri*) [128]. The authors compared the effectiveness of 12 types of edible oils and reported higher levels of xanthophyll extraction by short-chain (C4 and C6) triacylglycerides, medium-chain (C8) triglycerides, and fish oil compared with other edible oils, e.g., rice bran, rice germ, rapeseed, sesame, corn, soybean, and linseed. According to the study, fucoxanthin would be more stable in unsaturated edible oils, such as short-chain and medium-chain triglycerides, than in other edible oils containing polyunsaturated fatty acids. Kang & Sim used vegetable oils (soybean, corn, olive, and grape seed) and extracted astaxanthin from *Haematococcus* with the highest recovery of 93% with olive oil [129]. Karnila et al. optimized the duration of the solid–liquid extraction (SLE) of astaxanthin from vanname shrimp with palm oil and reached the highest yield value of 80% after 4 h extraction [130]. Astaxanthin was derived from *Haematococcus* and its stability in various edible oils was determined by Ranga Rao: this xanthophyll is stable at ambient temperatures [150]. The increase in the temperature above 70–90 °C leads to a reduction in astaxanthin content.

Edible oils extract total carotenoids [119,120,121,122,123,124,125]. These solvents are not selective for individual carotenoids. However, plants rich in specific carotenoids are its proper source. For example, lycopene was extracted in good quantities from tomato waste [127], fucoxanthine from edible brown seaweed [128], and asthaxanthin from *Haematococcus pluvialis* [129]. It is difficult to discuss the recoveries and draw conclusions, because of the scarce information reported. Better stability of the extract obtained could be achieved in unsaturated edible oils [128]. There is one big advantage—the extracts obtained can be directly applied in food processing.

- Using terpenes

Among the terpenes, D-limonene has proven to be a proper alternative solvent for the extraction of non-polar natural compounds like carotenoids [151]. Such examples include a few successful attempts at carotenoid extraction from plant materials (Table 2). Aissou et al. obtained better carotenoid quantities from carrots using D-limonene than n-hexane by maturation at room temperature for 1 h (94.8% vs. 78.1% carotenoid recovery) [131]. The identified carotenoids in the extraction obtained were β-carotene (61.7%), α-carotene (32.5%), lycopene (5.5%), and lutein (0.34%). Chemat-Djenni et al. compared the effectiveness of D-limonene and dichloromethane in lycopene extraction from tomato fruits [132]. The lycopene yield obtained using D-limonene was lower than that obtained by using dichloromethane (13.1% vs. 19.2%). Boukroufa et al. used D-limonene to extract carotenoids from orange peels and compared it with a traditional extraction using hexane [32]. They applied the UAE technique and observed that the carotenoid content was practically the same for both solvents, which indicates that traditional organic solvents can be replaced by limonene successfully.

The extraction of carotenoids using terpenes is not well explored. The terpenes used did not exhibit good selectivity for individual carotenoids. There are some advantages, such as the achievement of good recoveries, the fact that the extracts can be used in food products directly and that 90% of the extractant is recyclable [13].

- Using supercritical fluids (SFs)

Supercritical CO_2_ is a nonpolar solvent, compatible with the extraction of slightly polar compounds with a low molecular weight, such as carotenoids [152]. De Andrade et al. successfully applied SFE for the extraction of carotenoids from carrot peels and other vegetable wastes, such as the flesh and peels of sweet potato, tomato, apricot, pumpkin, and peach, as well as the flesh and wastes of green, yellow, and red peppers (Table 2) [134]. The authors achieved excellent results: 88–90% recovery of β-carotene mostly, and the polar carotenoids are in very small amounts. Generally, extraction using supercritical CO_2_ is selective towards non-polar β-carotene. This method has one disadvantage: a co-solvent, like ethanol [134,137] and/or vegetable oils [135,136], is necessary. The astaxanthin yield from *Haematococcus* was 78–87% using SC-CO_2_ and ethanol as a co-solvent [138,139,140] (Table 2). Machmudah et al. achieved the highest recovery of 78% using 5% ethanol as a co-solvent, and the amount of astaxanthin in the extract obtained was 12.3%, so there were more non-polar substances co-extracted [138]. Nobre et al. reported the successful use of SFE-CO_2_ with 10% ethanol for the extraction of astaxtanthin from *H. pluvalis* and analyzed the quality of the extract obtained: canthaxanthin, lycopene, and β-carotene were co-extracted [139]. Martínez et al. achieved a good recovery (80%) of total carotenoids extracted using SFE-CO_2_ with 20% ethanol from *Rhodotorula glutinis* yeast, after pulsed electric field pretreatment [142]. The main carotenoid obtained was torularhodin, which makes *R. glutinis* yeast its proper source. Derrien et al. applied supercritical CO_2_ with ethanol for the recovery of chlorophyll and lutein from spinach wastes and confirmed higher recovery of phytopigments (70% for lutein and 96% for chlorophylls) compared with the conventional extraction technique (e.g., acetone) [153]. There are many examples of the co-extraction of chlorophyll by SFE-CO_2_ when the plant source is rich in it [10].

Vigano et al. studied the selectivity of supercritical CO_2_ extraction. The authors obtained carotenoid extract free from other products, like fatty acids and tocopherols, in three stages while changing the conditions (temperature and pressure). In this case, the yield decreased [154]. Because of the non-polarity of the carotenoids, their extraction using supercritical CO_2_ has a high recovery and can be selective to them. Among the carotenoids, the selectivity depends on the vegetable source. Additionally, target compounds can be obtained with no traces of solvent and can be directly included in the final food product. SC-CO_2_ is environmentally friendly and non-toxic, but it requires a large investment for industry [109].

The recovery of carotenoids by supercritical fluid extraction is comparable to the recoveries of extraction by edible oils (Table 2). For example, astaxanthin from *Haematococcus* yielded 87% using SC-CO_2_ [140] and 93% using olive oil [129]. The extracts obtained using both approaches can be directly applied in the food industry without subsequent steps of enrichment or solvent removal. SFE can be selective to carotenoids in a gradient mode of extraction [154]. There are no data regarding the stability of the carotenoid extracts obtained by SFE.

- Using natural deep eutectic solvents (NADESs)

The use of choline chloride (ChCl)-based deep eutectic solvents as co-solvents for the extraction of carotenoids in Buriti fruit (*Mauritia flexuosa*) wastes was studied, and the recovery was 1043 mg/100 g DM [143] (Table 2). It was demonstrated that it did not increase the ethanolic extraction yield, because carotenes do not interact with the ChCl. In another study, with a different combination of components, ChCl and tartaric acid in apricots, it was reported that these solvents extracted 41 mg/100 g of the dry sample, while organic solvents extracted 11.5 mg/100 g [30]. Dharani et al. achieved 80% lutein purity (proven by HPLC) of the extract obtained using the combination of ChCl + glucose [145]. Silva et al. performed the extraction of lycopene from tomato waste using a hydrophobic eutectic mixture of DL-menthol and lactic acid by UAE [33]. The results showed an excellent capacity for extracting lycopene (145 mg/100 g) at the optimized condition. UAE is the technique chosen by Zhang et al. to extract astaxanthin from shrimp wastes [146]. The combination of ChCl and 1,2-butanediol in 1:5 mol/mol ratio and ultrasonication for 30 min at 20 kHz and 200 W resulted in a maximum yield of 0.15 mg/g and 0.22 mg/g xanthophyll extracted from shrimp shells and heads, respectively.

Stupar et al. optimized the extraction of carotenoids from pumpkin using NADESs based on fatty acids. The extracted carotenoids were β-carotene (70.2 µg/mL), β-cryptoxanthin (69.8 µg/mL), and lutein (1.9 µg/mL) [147]. After extract polarity switching, the precipitated fraction was obtained, which was richest in β-carotene (52%), followed by β-cryptoxanthin (38%), while the least abundant was lutein. The authors observed the stability of the extract obtained and reported 2.2% carotenoid degradation when the extract was kept for 1 month in the dark in a refrigerator, and 7.3% degradation occurred during a 6-month storage period. Referring to the obtained results, they concluded good stability of pumpkin carotenoids stored in NADESs as a solvent if kept in the dark at 4 °C for 6 months. The study reported that, due to the non-toxicity and biodegradability of the fatty acids, the purification step of the extract can be omitted, and the extract can be directly used in food.

The recoveries of carotenoids using NADESs were high: 76–96% (Table 2). Different techniques were applied: maceration, UAE, and MAE. The selectivity potential of NADESs depends on the combination of deep eutectic components [147]. The extract stability is high due to the unique intermolecular interactions between its components.

- Using ILs

In the literature, data about IL application for carotenoid extraction are scarce. Martins & de Rosso extracted lycopene from tomatoes using 1-butyl-3-methylimidazolium chloride-based ILs and compared their effectiveness with acetone [148]. The results showed greater yields by using 1-butyl-3-methylimidazolium chloride ([BMIM][Cl]). The total recovery of lycopene from tomatoes was 5.6 µg/g, compared to 3.7 µg/g with acetone (Table 2). Murador et al. developed a carotenoid extraction process from orange peels using ILs combined with an ultrasonic technique [149]. They tested four different ionic liquids and compared the extraction yield to that obtained using acetone. The most effective IL was [BMIM][Cl], with a total carotenoid extraction of 32 µg/g, as compared to the extraction yield of 7.8 µg/g obtained using acetone. The effectiveness of ILs can be explained by the affinity of [BMIM][Cl] to the carotenoid compounds due to the existence of hydrophobic and hydrogen bond interactions. Moreover, ILs disrupt the cellulose wall easily, which forces the extraction process.

To recover the carotenoids from ILs a few methods have been described, such as back-extraction and antisolvent precipitation The use of ion-exchange resins, microporous resins, distillable ILs, and thermo-responsive polymeric ILs are some other alternatives [13]. One example of a recovery process for orange peel carotenoids from ILs was developed by Murador et al. [149]. They used XAD-7HP resin with a recovery of the ILs from 59.5 to 63.8% and carotenoids from 52.2 to 58.7%, which raises the possibility of including the obtained extracts directly in food products as natural pigments. Nevertheless, to achieve better results and determine IL effectiveness, more research is needed to optimize the recovery process.

### 4.2. Extraction of Anthocyanins (ACNs)

ACNs are polar and water-soluble substances. The proper solvents are water and alcohol. Their extraction by polar solvents can be selective under some conditions. The polar BLNs could not be co-extracted, as both classes of natural colorants do not occur simultaneously in the same plant source [101]. However, non-phenolic substances, such as sugars, organic acids, and proteins, may be present in the extract and, hence, it is necessary to purify the extract [155]. Nearly 80% of process costs are associated with purification methods [156]. Purification can be achieved by membrane separation [157,158,159,160] or preparative chromatography [24,161].

The use of anthocyanins as natural food colorants poses several problems. They are not stable to light, oxygen, enzymes, metals, pH, and temperature [2,162]. ACNs with particular substituents on the flavyl nucleus (e.g., sugar groups esterified with phenolic acids) maintain better stability to heat and light [163,164]. As reviewed by Rodriguez-Amaya et al., suitable sources of acylated anthocyanins are radishes, red potatoes, red cabbage, black carrots, and purple sweet potatoes [2]. Their stability decreases at temperatures above 100 °C [10] and higher pH, but the beneficial effects of ACNs cannot be ignored [165]. A successful stabilization approach is the encapsulation of extracted ACNs [12,162].

The developed extraction methods using green solvents for extracting ACNs from plants for use as food colorants, separated into groups, are listed in Table 3.

- Using water

Water is the best “green” solvent. It can be used pure, without additives [166,167]. Usually, it is acidified [159,168,169,171,172,173] or/and mixed with ethanol [157,160,170,175], methanol, or acetone for ACN extraction from plants and foods [25]. Methanol and acetone are not eco-friendly. Acidification is necessary for the prevention of the degradation of non-acylated derivatives [12]. It is achieved by using hydrochloric, acetic, and/or phosphoric acid (Table 3).

Several techniques can be applied to solid–liquid extraction: maceration [172,175]; stirring [157,159,171,173]; UAE [167,169,170,174]. Some authors compared different extraction techniques based on their efficiency. Pinela et al. compared heat-assisted extraction and UAE of ACNs from *Hibiscus sabdariffa* calyces [174]. The extraction by UAE showed better results. Under optimized UAE conditions, the ACN yield was 24 mg/g DM (Table 3). Other compounds were also co-extracted.

The cavitation and mechanical effects produced by ultrasound may destroy the ACN structure in the process of UAE. Therefore, the ultrasonic conditions (ultrasound power, extraction temperature, solid-to-liquid ratio, and extraction time) must be strictly controlled [155]. Valencia-Arredondo et al. extracted red cabbage, firstly by subjecting it to acidified water at 24 °C for 12 h, and then it was subjected to the purification steps of micro, ultrafiltration, and adsorption processes [172]. Because of this, the pigment concentration was increased three times as compared to the initial content.

Diaconeasa et al. reviewed ACN recovery from food waste and byproducts [26]. The most widely applied method for this extraction used an acidified mixture of water and methanol, and the yields were scarce due to the decreased quantities caused by the previous food processing.

The use of acidified water combined with different extraction techniques leads to a higher stability of ACN in the extracts obtained. However, this approach is not selective in terms of these pigments, and purification is needed [155].

Fruit juices are easily acquired, and allow the extraction of different metabolites, including ACNs, thus, they have been proven to be a proper ACN source [12]. Another good alternative as an ACN source is some edible flowers [27]. The powder of dried and ground blossoms can be used directly as a food colorant. In this way, ACNs are protected in the plant cell. Extraction, purification, and stabilization can be avoided, which leads to economic benefits. However, the low stability must be considered, and the most appropriate storage conditions and the longest possible shelf life of dried ground plant material must be established.

- Using SF

The extraction method using SF as CO_2_ and H_2_O is characterized by a low treatment temperature and is especially suitable for the extraction of heat-sensitive substances, such as ACNs [179]. However, no SFE method has been developed for the selective extraction of ACNs.

King et al. applied SFE using acidified water (0.01% HCl, pH ~2.3) [176]. The process occurred at high temperatures, between 110 and 160 °C, and under a constant pressure of 40 bars. It is a highly efficient technique for the extraction of ACN from fruit. From haskap berry pulp, a 53% ACN extraction yield was obtained by supercritical CO_2_ extraction at optimum conditions of 65 °C, 15 min static and 20 min dynamic time, and 45 MPa [178]. The authors found that using water or ethanol as a co-solvent does not significantly change the yield. Seabra et al. developed a method for the SF-CO_2_ extraction of ACNs from the food residue elderberry pomace [182]. The researchers achieved the formation of an enriched extract in a two-step process: in the first step using SC-CO_2_ they obtained 5.2%, and in the second step using SC-CO_2_ and a mixture of ethanol/water in gradient mode they obtained 15% ACN in the extract.

The recovery of ACN increases using the SFE, and in gradient mode can be selective to these colorants, but not to individual ones.

- Using NADESs

More eco-friendly and with a high recovery is the extraction of ACNs using deep eutectic solvents. Several methods were optimized for NADES-based ACN extraction with better yields compared to an exhaustive extraction with the organic solvent. Moreover, on the one hand, the biodegradability of NADESs is high, and on the other hand, the acids in the solvent composition are effective in maintaining the color of the ACN extract [185]. NADESs represent an efficient green alternative to harmful organic solvents for the extraction and storage of ACN from plant materials [187]. The techniques applied are diverse: stirring [184,187]; UAE [158,161,185,186]; MAE [183,189,190] (Table 3).

*Hibiscus sabdariffa* was subjected to MAE at 550 W and 35 mL using citric acid/ethylene glycol with a 1:4 molar ratio to obtain the highest extraction of total ACN of approximately 3 mg cyanidin-3-glucoside equivalent/g [183]. The ACN extraction level reached was 1.24-fold higher using NADESs (6 mg/g FW) than using an organic solvent from mulberry [184]. Grillo et al. studied two green extraction methodologies, namely, MAE and UAE, for the recovery of ACNs from mulberry residues using five distinct NADESs [190]. Both technologies obtained superior performance in comparison with conventional extraction. MAE and UAE yielded 26 and 21 mg/g of total anthocyanin content, respectively.

Extraction by NADESs leads to the formation of more stable ACN extracts, which can be used directly in food coloring.

### 4.3. Extraction of Betalains (BLNs)

Commercial beet colorants are available as either juice concentrates (produced by vacuum-concentration of beet juice to 60–65% total solids) or powders (produced by freeze- or spray-drying), containing from 0.3% to 1% pigment [101]. However, there is a specific beet odor.

BLNs are water-soluble, like ACNs, but less stable than ACNs at temperatures above 25–30 °C [101]. BLNs are stable in the pH range of 4–5, with betacyanins (BCs) remaining unchanged for at least 20 days at 4 °C and over 275 days at −30 °C [191]. The plant sources never contain both groups of these pigments. Therefore, ACNs cannot be co-extracted with BLNs. Cai & Corke compared *Amaranthus* BCs and commercial colorants in terms of their color characteristics and stability at different temperatures in model food systems [192]. BCs exhibited a brighter red color than the red radish ACNs. Both pigments showed similar color stability at 14 °C and 25 °C, but the BC color was less stable than the red radish ACNs at 37 °C. A successful stabilization approach is the encapsulation of the BLNs extracted [162], or the addition of flavan into liquid BLN, which promotes the half-life and stability of BLN up to 60 °C [193].

The extraction of BLNs has more challenges than this process in ACN production from natural sources. The developed extraction methods using green solvents for betalains from plants as food colorants, separated into groups, are listed in Table 4.

- Using water

The most applied solvent is water, and the most used sources are beetroots (including beetroot waste), amaranth, and cactus fruits. Slight acidification enhances BLN stability and avoids oxidation by polyphenol oxidases [106].

Red beet is the most applicable source of BLNs for use as colorants in the food industry [2]. Therefore, beet varieties with higher BLN content are selected and grown. Sigwela et al. compared the efficiency of different water/alcoholic mixtures and techniques (stovetop and microwave) for BLN extraction from different varieties of beetroot and cactus pear fruits [209]. They also studied the stability of the extracts obtained. These studies reported that: the BLN yield was most influenced by the type of cultivar; the most heat-stable pigments were the red/purple pigmented fruit cultivars; all pigments were stable at pH = 4.5 and unstable at pH = 1; colors did not show substantial changes before and after UV-light exposure.

Sing et al. optimized the MAE process and concluded that pH is the most significant parameter [92] (Table 4). They achieved the best recovery of BLN from beetroot peels at pH 5.2 and by the use of citric acid. Borjan et al. compared different techniques (maceration, Soxhlet extraction, UAE, and SFE) and solvents for the extraction of BLN from beetroot [194]. Regarding the techniques, there were no significant differences in the recovery, except for SFE: in the extracts obtained by SE-CO_2,_ no betalains were detected. The scientific team concluded that water is the best solvent for BLN extraction, while SE-CO_2_ was not suitable for BLN extraction. Zin & Bánvölgyi did not detect a significant impact on the BLN recovery from beetroot peels using citric acid and microwave power [196]. They achieved the highest yield from MAE using only water, but citric acid increased the stability of the extracts obtained. Kumar et al. also demonstrated the satisfactory extracting potential of a citric acid aqueous solution using ultrasonication at pH = 5 [197]. Gómez-López et al. conducted a pressurized liquid extraction of polar biological active byproducts from *Opuntia stricta* var. Dillenii’s fruits [200]. The best recovery of betacyanins (betanin and neobetanin) was achieved under a constant pressure (10.34 MPa) at 25 °C in 50% ethanol. The extraction obtained was not selective towards beltalains. The researchers determined the concentration of phenolic acids and flavonoids in the extract obtained by HPLC.

The red beet betacyanin profile consists mainly of betanin and therefore offers a limited color range. It also possesses an undesirable flavor and carries the risk of soil-borne bacteria [210]. The alternative source of BLN, the cacti fruits, contain sugars, which were co-extracted. The sugar content decreases by using water/ethanol mixtures. Sanchez-Gonzalez et al. extracted BLN from cacti fruits and detected that the yield increased with increasing water percentage, but the selectivity was low because of the high sugar content [201] (Table 4).

Another alternative is the technique of aqueous two-phase extraction (TPE). Sandate-Flores et al. achieved 52.3% BC purity of the top phase using a polyethylene/phosphate system to extract BC from yellow pitaya [83]. In another study, the authors optimized aqueous TPE based on ethanol/KH_2_PO_4_/K_2_HPO_4_ to obtain low-sugar betacyanins extracts from *Escontria chiotilla* [202]. BLNs were collected in the top phase. The researchers detected higher yields of BX rather than of BC in the top phase and concluded that the ethanol–phosphate system could be applied for the separation of BC and BX.

Amaranth is a proper BC source, and, by using pure water as a solvent, an extract that is rich in pigment but low in sugar (compared to the cactus extracts) and free from fragrance substances (compared to the beetroot extracts) can be obtained. The applied techniques include solid–liquid extraction, UAE, and MAE (Table 4). Lopez et al. extracted BLN from amaranth using water as a solvent and suggested the best stability conditions: temperatures lower than or equal to 22 °C and a pH between 4 and 7 [203]. Howard et al. determined the best amaranth sources from 48 genotypes and optimized the solid-extraction conditions [204]. The extracts obtained were rich in BC. Roriz et al. extracted BC from *Gomphrena globosa* L. flowers using UAE with water, and, after lyophilizing or spray-drying, successfully incorporated the natural colorant into cookies [205].

According to the reviewed data, the extraction of BLN using water is not selective. The acidification of water leads to an increased stability of these compounds in the extract obtained. A selective option is aqueous TPE extraction [83].

- Using ILs

Rosa et al. developed a new method for the extraction of BLN from beetroot waste using thermos reversible aqueous biphasic systems composed of quaternary ammonium-based ionic liquids (ILs) and polypropylene glycol (PPG) [207] (Table 4). Using N-ethyl-N-methyl-N,N-bis(2-hydroxyethyl) bromide the researchers achieved an extraction efficiency of 95% for betalains. Under these conditions, chlorophyll was extracted simultaneously, but separated in the upper phase of the polypropylene. The pigments were removed from the solvents using affinity resins with high recoveries. In the same study, low to negligible toxicity of the solvents used was determined. Stability tests showed that better-preserved betalains are contained in the IL-rich phase, retaining about 90% and 40% of the initial concentration after 15 and 30 days, respectively. In contrast, after 15 days of storage in water, their concentration dropped by 40%, and after 30 days total loss was determined.

The extraction of BLNs by ILs must be expanded, due to the demonstrated better selectivity and stability of the pigments extracted [207].

- Using DES

In the scientific literature, there is a lack of methods for the extraction of betalains by DES. Hernández-Aguirre et al. reported a new DES method based on a mixture of magnesium chloride and urea [208] (Table 4). The authors also tested BLN stability in the DES extracts: BLNs achieved 75% stability when they were kept under visible light for 150 days and stored for 340 days in the dark.

DESs have once again been proven as promising extraction agents, and studies on their application in the extraction of BLNs need to be expanded.

## 5. Conclusions and Future Perspectives

Plants are an inexhaustible source of natural products, including pigments like carotenoids (yellow to orange), anthocyanins (red to blue), and betalains (red and yellow), which have found application as food colorants. These plant byproducts not only provide color to the food, but, as antioxidants, they can protect the food to which they are added, and can also apply functionality to the food, increasing its health-promoting potential.

Natural colorants are very sensitive to exposure to light, air, high temperatures, and chemicals. Stability and diversity in terms of color can be optimized by using environmentally friendly and selective extraction processes that provide a balance between the efficacy, safety, and stability of the resulting extracts. Green solvents, like water, supercritical fluids, natural deep eutectic solvents, and ionic liquids, are the most proper environmentally friendly solvents to be combined with different extraction techniques, like maceration, supercritical extraction, ultrasound-assisted or microwave-assisted extraction.

The first step to obtain higher yields is the selection of plant sources rich in the required pigment.

For extracting carotenoids, the proper green solvents are edible oils. Oils rich in unsaturated fatty acids stabilized carotenoid extracts better. These can be directly applied to food production. Applying SF-CO_2_ can lead to the selective extraction of carotenoids in gradient mode extraction. Additionally, target colorants can be obtained with no traces of solvent and can be directly included in the final food product.

For extracting ACNs, the proper green solvents are acidified water, SF-H_2_O, and SF-CO_2_. Using these solvents, high selectivity and stability were not achieved, except when using SFE in gradient mode. The most appropriate approach could be the application of powdered blossoms of edible flowers as food color additives. In this way, the anthocyanins are protected in the plant cell. Because extracting, purifying, and stabilizing the resulting extracts will be avoided, the latter procedure results in lower production costs. However, the low stability of ACNs must be considered, and the most appropriate storage conditions and the longest possible shelf life of dried ground plant material must be established.

ACNs have better thermo-stability than BLNs. Therefore, BLNs are used for the coloration of cold dishes like ice cream and cold drinks. The most proper source for obtaining BLNs is amaranth, obtained using water extraction. Acidification stabilized these extracts.

Generally, the best choice for colorant sources is food and agricultural industry residues and waste (peels, seeds, and pomace), which are rich in bioactive compounds including pigments. The best choice for green solvents for the extraction of all natural colorants from all plant materials is NADESs. They offer the best selectivity, stability, and ability for direct use in food production. Unfortunately, these solvents are not yet used on an industrial scale. Scientific studies must be carried out in this direction.

## Figures and Tables

**Table 2:** Extraction of carotenoids using green solvents.

Carotenoid	Source	Solvent	Extraction Technique	Yield/ Recovery	Reference
Edible oils					
Total carotenoids	Passion fruit peel (*Passiflora edulis*)	Olive oil Sunflower oil	UAE * 39 min, 47 °C, 30/100 mL ratio	1.2 mg/100 g DW	[119]
Total carotenoids	Pomegranate peels (*Punica granatum* L.)	Sunflower oil	UAE, 30 min, 51.5 °C, 0.10 g/mL ratio	0.6 mg/100 g DW	[120]
Total carotenoids	Peach palm peels (*Bactris gasipaes*)	Sunflower oil	UAE, 30 min, 35 °C, and 1528 W/m^2^	163 mg/100 g DW	[121]
Total carotenoids	Mango pulp	Flaxseed oil	High shear Dispersion	0.84 mg/100 g DW	[122]
Total carotenoids	Carrot waste	Flaxseed oil	MAE ** 9.39 min, 8.06:1 ratio, 165 W power	77.5%	[29]
Total carotenoids	Carrot waste	Flaxseed oil	Magnetic stirrer	3.46 mg/100 g DW	[123]
Total carotenoids	Pumpkin peel	Corn oil	MAE/ UAE 1:10 ratio	3.8/ 3.4 mg/100 g DM	[124]
Total carotenoids	Carrot pomace	Oleic acid	UAE; 350 W; 50 °C; 12.5 min; 39 mL/g ratio	16.3 mg/100 g DW	[125]
β-carotene	Mandarin epicarp waste	Sunflower oil	UAE, 60 min, 60 °C, 0.4 ratio	0.14 mg/100 g DW	[126]
Lycopene	Tomato waste	Sunflower oil	UAE, 70 W/m^2^ 10 min	92 mg/100 g DW	[127]
Fucoxanthin	Edible brown seaweed, (*Sargassum horneri*)	Edible oils (fish oil)	12 h solid- liquid extraction at 50 °C 1:2 ration	0.7 mg/mL	[128]
Asthaxanthin	*Haematococcus pluvialis*	Olive oil	Mixing at room temperature, 1:1 ratio	93%	[129]
Astaxanthin	Waste from Vanname Shrimp (*Litopenaeus* *vanname*)	Palm oil	SLE 4 h at 70 °C 1:2 (*w*/*v*) ratio	80% 509 mg/100 g DW	[130]
Terpenes					
Total carotenoids	Orange peels	D-limonene	UAE; 208 W/cm^3^, 5 min, 20 °C, 1/10 ratio	4%; 1.1 g/100 g DW	[32]
Total carotenoids	Carrots	D-limonene	Maturation, room temp., 1 h	95% 29 mg/100 g DW	[131]
Lycopene	Tomato fruits	D-limonene	Liquid-liquid extraction; 4:10 (*w*/*v*) ration + 15 mL water	13% 2.4 mg/100 g FW	[132]
Supercritical CO_2_					
β-carotene	Carrot peels	CO_2_ 5.5% (*v*/*v*) ethanol	SFE, 30 min, 59.9 °C; 15 g/min CO_2_;	80% 4.9 mg/100 g DW	[133]
β-carotene, Lutein, Lycopene	Pumkin	CO_2_ 10% (*v*/*w*) ethanol	SFE, 70 °C, 35 MPa, 1.5 mL/min CO_2_	74% 110 mg/g	[134]
Lycopene	Tomato	CO_2_ 5% (*w*/*v*) canola oil	SFE, 40 °C, 400 bar, 478 g/min CO_2_	6.6 mg/g DW	[135]
Lycopene	Tomato	CO_2_ 10% (*w*/*v*) hazelnut oil	SFE, 65°C, 425 bar, 230 g/min CO_2_	72.5%	[136]
Lycopene	Tomato peels	CO_2_ 14% (*v*/*v*) ethanol	SFE, 62°C, 450 bar, 3175 g/min CO_2_	33%	[137]
Astaxanthin	*Haematococcus pluvialis*	CO_2_ 5% (*v*/*v*) ethanol	SFE, 55 MPa, 70 °C, 3 mL/g	78%	[138]
Astaxanthin Canthaxanthine Lutein and β-carotene	*Haematococcus pluvialis*	CO_2_ 10% (*v*/*v*) ethanol	SFE, 300 bar, 60 °C, 3 mL/g	73% 85% above 90%	[139]
Astaxanthin	*Haematococcus pluvialis*	CO_2_ 2.5 mL/g ethanol	SFE, 43.5 MPa, 65 °C, 2.3 mL/g	87%	[140]
Fucoxanthin	*Sargassum horneri* (Turner) C. Agardh	CO_2_ 10% ethanol	SFE, 250 bar, 45 °C, 27 g/min CO_2_ 2 h	77 mg/100 g DW	[141]
Fucoxanthin	*Sargassum japonica* J.E. Areschoug	CO_2_ 10% ethanol	SFE, 250 bar, 45 °C, 27 g/min CO_2_ 2 h	41 mg/100 g DW	[141]
Total carotenoids (torularhodin)	*Rhodotorula glutinis* yeast	CO_2_ 20% ethanol	Sample pretreatment by pulsed electric fields SFE 50 MPa 80 °C; 0.8 g/min	80% 27 mg/100 g DW	[142]
NADES					
Total carotenoids	Buriti peels (*Mauritia flexuosa*)	Choline chloride (ChCl)/ethanol	30 min, 50 °C, 0.1/2 ratio	1043 mg/100 g DW	[143]
Lycopene β-carotene	Tomato pomace	ethyl acetate/ethyl lactate (30/70 *v*/*v*)	UAE, 20 min, 65 °C/ 100 mL/g ration	76% 395 mg/100 g DM	[144]
β-carotene	Apricot pulp	ChCl/L(+)tartaric acid (2:1)/Methanol 80:20	UAE, 10 min, 35 C/ MAE, 20 min, 52 °C	41 mg/100 g 76 mg/100 g	[30]
Lycopene	Tomato pomace	DL-menthol/lactic acid (8/1 mol ratio)	UAE, 10 min, 70 °C/ 120 mL/g ration	96% 145 mg/100 g DM	[33]
Lutein	Marigold (*Targetes erecta* L.)	ChChl/glucose	No data	80% 369 mg/g FW	[145]
Astaxanthin	Shrimp byproducts	ChCl/1,2-butanediol (1:5 mol/mol)	UAE; 20 KHz, 200 W, 30 min 15 mL/g ratio	0.15–0.22 mg/g	[146]
β-carotene; lutein; β-cryptoxanthin	Pumpkin	Octanoic acid/decanoic acid (3:1)	UAE, 60 °C, 10 min, 52.5 W/cm^3^ 7 mL/g ratio	0.14 mg/mL 15 mg/100 g DM	[147]
ILs					
Lycopene	Tomato fruits	1-butyl-3-methylimidazolium chloride ([BMIM][Cl])	No data	5.6 µg/g FW	[148]
Total carotenoids	Orange peels	1-butyl-3-methylimidazolium chloride ([BMIM][Cl])	UAE 1:3 (*w*/*v*) ratio; 5 min in 6 repetitions	32 µg/g FW	[149]

* UAE—ultrasound-assisted extraction; ** MAE microwave-assisted extraction.

**Table 3:** Extraction of total anthocyanins (ACNs) using green solvents.

Source	Solvent	Extraction Technique	Yield/ Recovery	Reference
Water				
Black rice	Water	34.7 °C, 80 min, 1:30 g/mL ratio	16 g/100 g	[166]
Black carrot pomace	Water	UAE *; 102.4 W; 50 °C; 1:3 ratio	61−74 mg/L	[167]
Red cabbage	1% HClaq + ethanol (1:1)	Stirring, 1 min, ambient temp. 1:2 ratio	391 mg/L	[157]
*Hibiscus sabdariffa* L.	15% acetic acid (*v*/*v*)	Stirring, 25 g/L ratio, ambient temp., 48 h	No data	[159]
Blueberry bagasse	Acidified water	100 °C, 5 min	75% 17 g/100 g	[168]
Fresh purple eggplant parts	Acidified water	UAE, pH 2.0, 60 °C, 60 min 30 g/mL ratio	29 mg GAE/g DM	[169]
Bilberry	60% ethanol aq	pH 2.0 (HCl), 1:3 ratio; 50 °C, 1 h, 2 cycles	3.5 mg/mL	[160]
*Prunus spinosa* L. Fruit Epicarp	Water/ ethanol (1:1)	UAE, 400 W, pH 3; 50 g/L ratio, 5 min	18.2 mg/g DM; 68.6%	[170]
Campbell Early grape	Acidified by acetic acid water	Stirring, pH 2, 80 °C, 10 min, 1/50 g/mL ratio	198 mg/100 g DW	[171]
Red cabbage	Acidified by phosphoric acid water	24 °C, 12 h	32.5 mg/mL	[172]
Purple passion fruit peel	Potable water	Stirring, pH 2.0, 52 °C, 180 min 30 g/mL ratio	577 mg/100 g DW	[173]
*Hibiscus sabdariffa* L.	39.1% ethanol in water, *v*/*v*	UAE, 296.6 W, 26 min; 30–35 °C; 30 g/L ratio	24 mg/g DM	[174]
Egg plant (*Solanum melongera* L.)	50% ethanol aq	Maceration, 4 h 30 °C	62 mg/100 g	[175]
Supercritical fluid (SF)				
Fruit berries and their pomace	H_2_O	110–160 °C; 4 MPa; 4 mL/min (0.01% HCl, pH ~2.3)	90%	[176]
Blueberry	H_2_O	190 °C; 1 min	0.7 mg/g DW	[177]
Chokeberry	H_2_O	130 °C; 3 min	0.5 mg/g DW	[177]
Haskap berry pulp	CO_2_	65 °C, 15 min static and 20 min dynamic,45 MPa, co-solvent—water (5.4:3.2 *w*/*w* ratio)	53%	[178]
Blueberry	CO_2_	40 °C, 28 MPa, ratio 1:7 g/mL, 60 min	2 mg/100 g	[179]
Blueberry	CO_2_	40 °C, 20 MPa, and 10 mL/min	85 mg/100 g	[180]
Blueberry pomace	CO_2_	40 °C, pressure 34.7 MPa, 4.5 L/min, 1.86 h	1.5 mg/g	[181]
Elderberry pomace	CO_2_	39.85 °C, 21 MPa Two step extraction Ethanol/water (0.5–100/0–95%*v*/*v*)	15%	[182]
NADES				
Raspberry	ChCl/1,4-butanediol (1:3)	UAE; 29% water, 210 W, 51 °C, 32 min	1.4 mg/g	[158]
*Hibiscus sabdariffa*	Citric acid/ethylene glycol with a 1:4 M ratio	MAE; 550 W 50% water (*v*/*v*), 180 s	3 mg/g DW	[183]
Mulberry	ChCl-citric acid-glucose (1:1:1)	high-speed homogenization; 30% water, 22 mL/g, ratio 30 min	6 mg/g FW	[184]
Black carrots	ChCl;citric acid (1:1 mol/mol)	UAE; 78.4 W, 19.8 min, 24.5:1 mL/g ratio	17 mg/g	[185]
Bilberry	ChCl:sorbitol (1:1)	UAE; 34.8% water, 320 W, 48.4 °C, 10 mL/g ratio	0.3 mg/g DW	[186]
*Catharanthus roseus*	Lactic acid–glucose 1,2-propanediol–choline chloride	Stirring, 40 °C; 30 min	No data	[187]
Blueberry wine residues	Choline chloride + 1,4-butanediol (1:3)	UAE; 29% water, 380 W, 55 °C, 40 min	9 mg/g	[161]
Wine lees	ChCl/Malic acid	UAE, 30.6 min, 341.5 W, 35.4% water (*w*/*w*); 33.3 mg/mL ratio	6 mg/g DW	[188]
Grape-pomace	ChCl-citric acid (2:1)	MAE ** 300 W, 10 min, 30% water, 30 g/L ratio	0.6 mg/g	[189]
Grape-pomace	ChCl-citric acid (1:1)	UAE, 40 kHz, 50 W, 10 min	0.3 mg/g	[189]
Blueberry peel	ChCl-lactic acid (1:1))	MAE/UAE 22% water	26/21 mg/g	[190]

* UAE—ultrasound-assisted extraction; ** MAE microwave-assisted extraction.

**Table 4:** Extraction of betalains (BLNs) using green solvents.

BLN	Source	Solvent	Extraction Technique	Yield/ Recovery	Reference
Water					
Total BC	Moroccan prickly pear fruits (*Opuntia ficus indica)*	Water/ethanol/metabisulfite sodium (0.5%)	Stirring, 30 min, 4 °C	38 mg/kg yellow pear 46 mg/kg red pear	[84]
Total BLN	Beetroot peels (powdered)	Water acidified by citric acid	MAE *, 0.2 g/mL ratio; 224.61 MW 57.06 s; pH 5.2	472 mg/L	[92]
Total BC Total BX	Beetroot (*Beta vulgaris* L.)	Water	Soxhlet, 7.5 g/mL ratio; 3 × 2 h	3.8 mg/100 g 2.4 mg/100 g	[194]
			UAE **, 40 kHz, 40 °C, 1.5 h	2.8 mg/100 g 3.3 mg/100 g	
			Maceration, 2 h	3.6 mg/100 g 3.8 mg/100 g	
Total BC Total BX	Beetroot waste	Water	MAE, 800 W, 150 s; 0.2 g/mL ratio	172 mg/L 232 mg/L	[195]
Total BC Total BX	Beetroot peels	Water	MAE, 800 W, 150 s; 0.2 g/mL ratio	116 mg/100 g FW 86 mg/100 g FW	[196]
Total BC Total BX	Beetroot powder (freeze-dried)	Water acidified by citric acid	UAE, 10 min, 30 °C, pH = 5	4 mg/g DW, 3.6 mg/g DW 90% total BLN	[197]
Total BC Total BX	*Beta vulgaris* L. waste stalks	Water	UAE, 79.801 W/cm^2^ 26.7 min, 22.4 g/mL	3 mg/g 4.4 mg/g	[198]
Total BC	Dragon fruit peel (powdered)	Water	MAE; 35 °C; 8 min 40 mg/mL ratio	9 mg/L	[199]
BC	*Opuntia stricta* var. *Dillenii* fruits	Water/ ethanol 1:1 *v*/*v*	PLE; 10.34 MPa; 25 °C; 10 min	2.3 mg/g DW betanin 2.3 mg/g DW neobetanin	[200]
Total BLN	*Opuntia joconostle* cv.	Water/ethanol	30 °C; 30 min	86 mg/100 g	[201]
Total BX	Yellow pitaya (*Stenocereus pruinosus*)	Water	Aqueous two phase system (PEG/KH_2_PO_4_/K_2_HPO_4_) Mixing, 15 min; 25 °C; 10% *w*/*w* ratio	52%	[83]
BLN	Cacti fruit (*Escontria chiotilla*)	Water	Aqueous two phase system (Ethanol/KH_2_PO_4_/K_2_HPO_4_) Mixing, 15 min; 25 °C; 10% *w*/*w* ratio	63%	[202]
Total BC Total BX	*Amaranthus hypochondricus* L.	Water	Solid-liquid extraction	0.2–0.4 mg/g 0.2–0.6 mg/g	[203]
Total BC	Amaranth (*A. cruentus*)	Water	Solid-liquid extraction	4.8 mg/g	[204]
Total BC	*Gomphrena globosa* L. flowers	Water	UAE; 500 W, 22 min; 5 g/L ratio	161 mg/g DM	[205]
Total BC Total BX	*Amaranthus tricolour* leaves	Water	MAE, 400/250 W, 15 min	72 mg/g DW 42 mg/g DW	[206]
ILs					
BLN	Beetroot stems and leaves	Water/N-ethyl-N-methyl-N,N-bis(2-hydroxyethyl)bromide/PPG	Vortex; 20 °C, 70 min; 0.12 g/mL ratio	6.7% *w*/*w*	[207]
DES					
BLN	Beetroot	MgCl_2_ × 6H_2_O/Urea (2:1)	Stirring	4 mg/g	[208]

* MAE microwave-assisted extraction;** UAE—ultrasound-assisted extraction.

## Data Availability

The data presented in this study are available in this article.
